# Mental health and Multiracial/ethnic adults in the United States: a mixed methods participatory action investigation

**DOI:** 10.3389/fpubh.2023.1286137

**Published:** 2024-01-11

**Authors:** Jaimie Shaff, Xinzi Wang, Janel Cubbage, Sachini Bandara, Holly C. Wilcox

**Affiliations:** Johns Hopkins Bloomberg School of Public Health, Baltimore, MD, United States

**Keywords:** mental health, public health, racial groups, diversity, equity, inclusion, protective factors, risk factors

## Abstract

**Introduction:**

Addressing gaps in the integration of justice, diversity, equity, and inclusion (J-DEI) in public health research and practice, this study investigates the mental health of Multiracial and multiethnic adults in the United States (U.S.). A rapidly growing racial/ethnic group in the U.S., Multiracial and multiethnic populations are often excluded or underrepresented in standard public health research and practice, and little is known about their mental health or associated risk and protective factors.

**Methods:**

To investigate this knowledge gap, an electronic cross-sectional survey was conducted in two waves in 2022, pulling from various community sources, with 1,359 respondents in total. Complementing this, seventeen semi-structured interviews were performed with a subset of survey participants. Data were analyzed using a mix of statistical methods and staged hybrid inductive-deductive thematic analysis.

**Results:**

Findings indicate over half of the participants endorsed at least one mental health concern with prevalence of anxiety, depression, post-traumatic stress disorder, and suicidal thoughts and behaviors surpassing available national estimates. Exposure to trauma, discrimination, and microaggressions were found to play a significant role in these outcomes. Conversely, strong social support and strong ethnic identity emerged as protective factors. Qualitative insights brought forward the challenges faced by individuals in navigating bias and stigma, especially in the context of mental health care. Despite these barriers, emerging themes highlighted resilience, the importance of secure identity formation, and the critical role of community and cultural support.

**Conclusions:**

The marked prevalence of mental health concerns among Multiracial and multiethnic populations emphasizes the pressing need for tailored interventions and inclusive research methodologies. Recognizing and addressing the unique challenges faced by these communities is imperative in driving mental health equity in the U.S. The findings advocate for community-engaged practices, interdisciplinary collaborations, and the importance of addressing mental health challenges with cultural sensitivity, particularly in historically oppressed and marginalized groups. Future efforts must focus on refining these practices, ensuring that public health initiatives are genuinely inclusive and equitable.

## 1 Introduction

Until 2020, Multiracial and multiethnic people, or people who identify with two or more racial and/or ethnic groups, were thought to be a relatively small proportion of the United States (U.S.) population. Data on the health status of people with two or more races and/or ethnicities were infrequently reported in national data surveillance reports and scientific literature, and studies among Multiracial and/or multiethnic populations often included small samples with limited generalizability. However, information from the U.S. Census Bureau in 2020 illuminated a stark undercount of Multiracial people: following a slight change in question structure and coding procedures, the U.S. Census Bureau calculated a 276% increase in the population between 2010 and 2020 (1). While part of this increase is attributable to population growth, we can estimate the impact of this classification change on population estimates by using the American Community Survey (ACS) population estimates for 2019 and 2021: the 2019 ACS estimated people with two or more races to make up 3.5% of the population, versus 12.6% as estimated in 2021 (2). When comparing these two population estimates, we also see that the proportion of each generation that is Multiracial increased from 1.4 to 7.7% for Baby Boomers, 2.3 to 11.7% for Gen-X, 3.5 to 12.9% for Millennials, and 5.8 to 16.6% for Gen-Z (2). This is likely an undercount as the current approach has limited inclusion of people identifying as Hispanic/Latino, requires individuals to identify with available categories, and uses a measurement approach that has resulted in low estimates of the Multiracial and/or multiethnic population when compared to other approaches (3–6).

Such discrepancies in data representation do not merely skew demographics but, more alarmingly, they directly influence public health strategies, resource allocation, and interventions. The elimination, erasure, misclassification, and concealment of populations from public health data perpetuates structural racism and impacts progress toward health equity (7, 8). Before and throughout the COVID-19 pandemic, data have been used to drive decision making, allocate resources, and monitor health inequities. However, even after over 100 million documented cases and 1 million deaths, the Centers for Disease Control and Prevention (CDC) COVID-19 Data Tracker continues to exclude cases for people with multiple races (9). This results in Multiracial/ethnic populations remaining invisible and not receiving the attention and resource allocation from policies and programs that could reduce disparities and improve health and wellness.

The U.S. has a complicated history with Multiracialism. Examples such as the “one-drop rule,” a legal classification system in the U.S. requiring anyone with a single ancestor with Black heritage to be classified as Black, and anti-miscegenation laws, which banned marriage and sometimes even intimate relations between people of different races, demonstrate the complex legal history for Multiracial and multiethnic communities within the U.S. There is additional complexity within U.S. military history, with notable examples from World War II and the Vietnam War of mixed-race children fathered by U.S. soldiers during military involvement overseas. Unfortunately, the very existence of Multiracial and multiethnic people continues to be challenged in the U.S. The sociopolitical decisions resulting in the many ways the U.S. Census Bureau has collected data on race/ethnicity are a clear example of systemic racism (10). Before the U.S. Census Bureau added the ability to select more than one race in the 2000 Census, there was much debate and controversy about the need, legality, or utility of quantifying the Multiracial population (11). Even more recently, although state laws banning miscegenation were deemed unconstitutional in 1967 by the Supreme Court decision Loving v. Virginia, a rise of social movements against miscegenation required the U.S. to pass a law providing federal protection for interracial marriages in 2022 (12). A historic event, the social context necessitating federal protections for interracial marriages gives some indication of the current environment.

Such chronic societal stresses can lead to “weathering,” a phenomenon where individuals consistently coping with race-related stress experience detrimental health outcomes (13). Evidence suggests weathering is exacerbated among individuals belonging to fewer social groups and individuals with multiple structurally disempowered identities, while strong ethnic identity can serve as a protective factor (14–16). Research conducted before and during the COVID-19 pandemic has elucidated mental health disparities with more rapidly increasing rates of anxiety, depression, and suicidal thoughts and behaviors (STB) in historically marginalized groups (17–20). There has also been increased attention to the impact of adverse experiences, including trauma and discrimination, on health (21–23). Associations between minority stress and mental health have been well established for monoracial populations, yet are lacking for Multiracial and multiethnic populations (24–26). A 2023 article described the impacts of experiences unique to the Multiracial and multiethnic community, such as rejection from monoracial communities and “monoracism,” or discrimination for being a member of multiple groups, and the importance of social support and multiethnic identity integration for this diverse population (27).

As we emerge from the acute phases of the COVID-19 pandemic, the U.S. is grappling with a mental health crisis (28). Unfortunately, data on the mental health status of Multiracial and multiethnic populations are rarely reported out in national or local public health surveillance efforts or in scientific research. During the COVID-19 pandemic, information from the Centers for Disease Control and Prevention’s Household Pulse Survey provided critical data updates on the mental health status of the population: the racial group that combined people who identify as non-Hispanic and either “Other” or as “Multiple Races” consistently had the highest prevalence of symptoms of anxiety and/or depression compared to other racial groups for almost every time point (29). For example, in a recent round of data reporting (July 26–August 7, 2023), the prevalence of symptoms of anxiety disorder or depressive disorder was 43.3% (95% CI: 39.5–47.1) for respondents identifying as non-Hispanic, other races and multiple races; 36.7% (95% CI: 34.2–39.1) for Hispanic or Latino, 32.5% (95% CI: 30.2–34.8) for non-Hispanic Black, single race; 31.9% (95% CI: 31.1–32.6) for non-Hispanic White, single race; 23.2% (95% CI: 20.3–26.2) for non-Hispanic Asian, single race. However, combining “Other” and “Multiple Races” into a single category, paired with the known challenges with capturing racial and ethnic data, limits the utility of these data to drive public health action and resource allocation.

In recent years, several studies have suggested Multiracial and multiethnic people could have some of the highest rates of mental health concerns out of any other racial or ethnic group. Studies among adolescent and young adult populations identified Multiracial youth to have poorer mental health than monoracial youth (30–32). In August 2022, the Trevor Project released “The Mental Health and Well-Being of Multiracial LGBTQ Youth,” a report that found that Multiracial lesbian, gay, bisexual, transgender, queer or questioning (LGBTQ) youth reported more mental health challenges than monoracial peers (33). Several months later, the Substance Abuse and Mental Health Services Administration (SAMHSA) released data from the 2021 U.S. National Survey of Drug Use and Health (NSDUH) with detailed information by race and ethnicity. For what appears to be the first time, NSDUH provided specific data for Multiracial populations as compared to other racial and ethnic groups: Multiracial adolescents had the highest proportion of past year major depressive episode (27.2%) and Multiracial adults had the highest proportion of any mental illness (34.9%) and serious mental illness (8.2%) (34). These two reports provide concerning indications of mental health inequities experienced by Multiracial and multiethnic populations and provide concerning indications of the impacts of the absence of consistent, high quality, actionable public health data on mental health for Multiracial and multiethnic populations across the U.S., let alone for individual localities. Despite these data becoming available over recent months, these reports lack information on risk factors, protective assets, or unique health needs of this diverse community. Additionally, few studies have assessed mental health among older Multiracial and multiethnic adult populations and a 2019 review of studies from 1990 to 2009 highlights methodological challenges and inconsistency across studies, limiting comparability of results and understanding of the growing Multiracial population (4). With millions of federal dollars being allocated to address mental health in the U.S., the limited visibility of mental health among Multiracial and multiethnic populations could impact resource allocation; the SAMHSA Office of Behavioral Health Equity does not list “Multiracial” as a priority population (35, 36).

The increasing rate of mental health concerns among historically excluded and underrepresented groups, especially during and post-COVID-19 pandemic, and the unique stressors associated with being Multiracial and multiethnic, underline the imperative need to promote research centered around justice, diversity, equity, and inclusion (J-DEI) in mental health and substance use research. This is crucial, not only for the sake of accurate representation but for the well-being of a significant and growing segment of the U.S. population. For Multiracial and multiethnic people, mental health is complex and dynamic. Aspects of language, culture, and history influence mental health (37). Acculturation mismatch, intergenerational culture conflict, historical trauma, perceived discrimination, and racism are associated with adverse mental health outcomes that impact racial and ethnic groups in different ways (21, 38–41). A recent article highlights the unique experiences of discrimination, social exclusion, and ethnic identity formation experienced by Multiracial and multiethnic people (27). With the Multiracial and multiethnic demographic being the fastest-growing racial group in the U.S., there’s an urgent requirement to understand the complexities of their mental health challenges (1).

The aims of this study are to (1) illuminate the mental health landscape of Multiracial and multiethnic adults in the U.S., emphasizing the disparities and unique challenges they face, (2) highlight the impact of data erasure, exclusion, and underrepresentation in public health systems, urging for more inclusive methodologies, and (3) advocate for a proactive shift in public health research and practice, integrating J-DEI principles, to bridge critical knowledge gaps and drive mental health equity.

## 2 Methods

### 2.1 Sample populations

The Johns Hopkins Bloomberg School of Public Health Institutional Review Board (IRB) approved this study (IRB Protocol 18,482). Two nonprobability-based convenience samples were obtained through an online anonymous survey collected from February to June 2022 (Sample A) or October 25 to December 6, 2022 (Sample B). Participants were eligible to participate in the study if they were 18 or older, lived in or were from the U.S., confirmed high level of ability in reading the English language, identified as Multiracial and/or multiethnic, and identified with two distinct categories from eight available options for racial/ethnic identity (White, Black or African American, Asian, Native Hawaiian or Pacific Islander, American Indian or Alaska Native, Middle Eastern or North African, Hispanic or Latino, Other). Respondents for Sample A were not compensated and were recruited through social media (Facebook, Reddit, Twitter, LinkedIn), organizations and groups supporting Multiracial and multiethnic people, public health listservs, and ResearchMatch. In an effort to achieve a larger sample size, respondents for Sample B were recruited from multiple market research panels facilitated by Qualtrics, which aims to mirror census representation, and compensated up to $9.50 (42). To participate in this anonymous study, non-identifying informed consent involved participants individually verifying they met each of the eligibility criteria, including being at least 18-years of age. They were then required to read the consent document, acknowledge that they read and understood it, and consent to participate in the study. A subset of participants from the cross-sectional studies were invited to participate in semi-structured interviews. Interviews were conducted via videoconference or audio call from December 2022 to January 2023 with adults 18 or older who identified as Multiracial and/or multiethnic, live in or are from the U.S., and endorsed at least one symptom of a mental health condition in the survey. Informed consent was obtained verbally. For participating in the qualitative interview, participants received a $25 Amazon gift card as a small token of appreciation. Participants were provided with contact information for both the study team and the IRB and were provided with information for crisis help lines at the beginning and end of the survey, within informed consent materials, and in each correspondence related to the interview.

### 2.2 Participatory action approach

These studies were conducted with the principles of participatory action research (PAR), a powerful approach to research and practice that serves to shift the power balance back to the populations of focus to inform action (43). Led by the community of focus, PAR systematically includes the population being researched at every level of research and results in a research design that utilizes community-responsive methods and analytic approaches, with action-oriented findings developed by and for the community (44–46). Led, guided, and championed by the community of study, participatory approaches can explore and address the cultural challenges of assessing mental health status within diverse populations. This is an essential public health activity as tools typically used to assess mental health status may not translate properly with all cultures and PAR approaches provide the opportunity to integrate known and unknown cultural norms and practices within the study design (47). Studies have demonstrated the clinical utility of idioms of distress, which vary by culture and context and may not be widely recognized by healthcare providers (48). Assessing the mental health needs of multicultural individuals, who are known to adjust to different environments, presents additional challenges (49, 50). The American Public Health Association recommends the use of participatory approaches by leaders from the impacted community to achieve meaningful progress toward health equity (51). As examples for how the authors utilized PAR throughout this body of work, the lead author identifies as Multiracial and multiethnic and was joined by an advisory group of three individuals who identify as Multiracial and/or multiethnic that participated in developing the study design and final instruments. Qualitative data in were analyzed by two analysts who identify as Multiracial and/or multiethnic. Participants from the qualitative study were invited to review and provide comments on the preliminary findings; these recommendations were incorporated.

### 2.3 Definition of Multiracial and multiethnic

National standards for racial and ethnic data collection set by the federal government include five categories for race (American Indian or Alaska Native (AI/AN), Asian, Black or African American, Native Hawaiian or Other Pacific Islander, White, Other) and two categories for ethnicity (Hispanic or Latino, not Hispanic or Latino) (52). To the authors’ knowledge, most public health entities and scientific research studies do not incorporate Hispanic and Latino populations within their definition of Multiracial. As this study includes Hispanic and Latino populations as well as populations identifying as “Middle Eastern or North African,” this study refers to the sample population broadly as Multiracial and/or multiethnic. Racial categories are socially constructed and have changed throughout history (53, 54). Scholars argue that these seven groups are distinct racial groups due to their uniquely racialized experiences, and this is reflected in the drafts for the updated 2030 census format, which no longer asks separately about Hispanic ethnicity but include all of seven groups in one question (53). Please note that this approach to gathering racial/ethnic background does not fully or adequately capture the rich diversity of the study population, nor the language used by each individual participant to describe their background.

### 2.4 Measures

Mental health symptoms were assessed using several instruments validated for self-reported symptoms of psychiatric conditions. Depressive symptoms were assessed using the 9-item Patient Health Questionnaire (PHQ-9), a validated tool based on the DSM-5 criteria, allowing clinicians to grade the severity of a patient’s symptoms (55). Symptoms of anxiety were assessed using the 2-item General Anxiety Disorder Scale (GAD-2) (56). For each of the nine items of the PHQ-9 and the two items of the GAD-2, participants were asked to provide a response on a 4-item Likert Scale (0: Not at all, 1: Several Days, 2: More than half the days, 3: Nearly every day) how often they had been bothered by the item over the past weeks. Symptoms of post-traumatic stress disorder (PTSD) were assessed using both the Life Events Checklist for DSM-5 (LEC-5) and PTSD Checklist for DSM-5 (PCL-5), tools validated for self-assessments (57, 58). The LEC-5 is designed to assess exposure (personally experienced, witnessed, learned about it, exposed as part of one’s job) to 16 potentially traumatic events over their lifetime. To be classified as having clinically significant symptoms of PTSD, a participant must have endorsed exposure to at least one item on the LEC-5. The PCL-5 is a 20-item instrument that asks participants to provide a response on a 5-item Likert Scale (0: Not at all, 2: A little bit, 3: Moderately, 4: Quite a bit, 5: Extremely) of how much the participant had been bothered by each problem in the past 30-days. History of suicidal thoughts and behaviors (STB) were assessed using five items from the National Survey on Drug Use and Health that ask the respondent to provide a response (Yes, No, Prefer Not to Say) if they had experienced each STB in the past 12 months (34).

To better understand the context of individuals participating in the study, the survey collected information on social support, stress, discrimination, harassment, and microaggressions. Self-reported stress was measured by the Perceived Stress Scale (PSS-4), a widely used, validated instrument for measuring non-specific perceived stress (59). Exposure to discrimination was measured through the two subscales of the Perceived Discrimination Scale to establish exposure to up to 11 types of Lifetime Discrimination and volume of exposure (maximum score of 36) to Daily Discrimination; this instrument has been previously tested among racially diverse populations (23, 60). Exposure to 45 different microaggressions was assessed using the Racial and Ethnic Microaggressions Scale (REMS), an instrument designed to measure these experiences among people of color (61). Perceived social support was assessed using the 12-item Multidimensional Scale of Perceived Social Support (MSPSS), a tool validated for use within a non-White population (62, 63).

Strength in ethnic identity was measured across three domains (exploration, resolution, affirmation) of the 17-item Ethnic Identity Scale (EIS) (64). Exploration had a maximum possible score of 28, resolution 16, and affirmation 24; higher scores indicated greater strength in each domain of ethnic identity. Multicultural identity integration was measured across three domains (categorization, compartmentalization, integration) of the 22-item Multicultural Identity Integration Scale (MULTIIS) (65). Categorization had a maximum possible score of 35; compartmentalization, 63; and integration, 56; higher scores indicated stronger configuration of each domain of multicultural identity. For example, higher scores in categorization and compartmentalization suggest a respondent endorses more separation of their cultural identities while higher scores in integration suggest a respondent endorses more blending of their cultural identities. The survey also collected demographic data on race and ethnicity, gender identity, sexual orientation, age, place of birth, educational attainment, and household income level.

A semi-structured interview guide was developed to explore attitudes and practices related to mental health, perceived barriers to achieving optimal mental wellness, concepts of resilience, and opportunities identified by the participants for improving mental health services for Multiracial communities. Intersectionality was explored to assess the added impact of colorism, gender, sexuality, age/generation, disability status, and socioeconomic status among Multiracial communities.

### 2.5 Strengths and limitations

This study has several strengths. One of the standout attributes is its utilization of diverse sampling methods to demonstrate the potential to rapidly assess the health status and needs of an underrepresented population. By employing both a social media-driven approach and one that mirrors the U.S. census, the research attempts to provide a holistic picture. Additionally, the inclusive approach taken by integrating Hispanic and Latino Multiracial and multiethnic populations offers an enriched perspective, broadening the scope of Multiracial categories. As the study utilized mental health instruments commonly used in clinical and community settings as diagnostic screening assessments and for psychiatric epidemiological research, we can compare our findings to national samples and other studies using these instruments. The adaptability of this study provides ample opportunity for public health practitioners to rapidly assess and respond to the unique needs of communities excluded/underrepresented by current infrastructure.

The study is not without limitations. Primarily, the cross-sectional nature of the research, which focused on English-speaking Multiracial and multiethnic adults with internet access, limits the generalizability of the results, comparability to monoracial groups, ability to establish temporality, and may not be reflective of the general population of Multiracial and multiethnic adults. As we included Hispanic and Latino Multiracial and multiethnic people in our study population, our results may have limited comparability to data that exclude Hispanic and Latino people from Multiracial categories. As the survey assessed mental health and took place following and during potentially traumatic global pandemics of COVID-19 and racialized violence, as well as mass social movements targeting minoritized communities, all known to have increased mental health concerns in the general population, the sample population may express a higher volume of mental health concerns than pre-pandemic times and has limited comparability to studies using data from prior decades. This study was not designed to assess differences over time. Additionally, a common challenge with mental health studies is the varying recall period. This study utilized the recall proposed by the instruments themselves and accepted a varying recall across mental health indicators ranging from 2 weeks to past year, and exposure to potentially traumatic and prejudice events ranging from daily to lifetime. Although selected instruments demonstrated reliability and validity within diverse populations, to the authors’ knowledge few instruments have been psychometrically tested within Multiracial and multiethnic adult populations in the U.S. As the qualitative study was limited to participants of a previously administered cross-sectional study who endorsed at least one mental health symptom and agreed to participate in a follow-up interview via videoconference or phone, the results have limited generalizability to Multiracial and multiethnic adults as a whole. Subgroup analyses were dependent on the demographics of the participants of this phase of the study. Additionally, this study is limited to participants who are alive at the time of the study and does not capture the experiences of people who did not survive a behavioral health crisis.

### 2.6 Data analysis

Statistical analyses were performed using STATA/SE 17.0. Self-reported mental health status was assessed by computing the scores for each mental health outcome according to the instrument’s guidelines. Severity was ascribed based on the results of the instrument. Endorsement of mental health conditions was established using cutoffs for clinically significant symptoms of anxiety (GAD-2 score ≥ 3), depression (PHQ-9 score ≥ 10), PTSD (exposure to at least one event on LEC-5 & PCL-5 score ≥ 33) or endorsement of any of the five measures for STB.

Respondents were categorized into racial and ethnic groups based on their self-reported racial and ethnic identity to assess differences within the Multiracial and multiethnic population. As this survey is for people who identify as Multiracial and multiethnic, ethnicity was incorporated with race as a component of multiethnic identity. Categories for Multiracial and multiethnic people with White and Non-White and Non-White racial/ethnic identities were developed to explore findings as compared to prior research, and to assess within-group differences (31). Differences by racial and ethnic heritage were further explored by categorizing the population into populations with any White, Black or African American, Asian, Native Hawaiian or Pacific Islander, American Indian or Alaska Native, Middle Eastern or North African, or Hispanic or Latino identity. Domains of exploration, affirmation, and resolution of the EIS and domains of categorization, compartmentalization, and integration of the MULTIIS were computed as continuous variables according to each instrument’s guidelines.

Adverse experiences were constructed as linear variables. Potentially traumatic experiences, lifetime discrimination, and microaggressions were computed by calculating the number of different situations experienced. Everyday discrimination was computed by summing the total of the Likert scale responses for the Daily Discrimination subscale. The differences between the different coding approaches to establish exposure to prejudice events will not be discussed in this paper, but are worth noting for future research (66). Perceived stress was viewed both continuously and then tested as a binary variable with the cutoff ≥6 indicating high levels of stress based on recent population norms; there is no established cutoff for the PSS-4 (67).

Variables were constructed for age, gender identity, sexual orientation, socioeconomic status, and place of birth as potential confounders or effect modifiers. As Multiracial and multiethnic populations have increased over time and rates of depression and STB have increased in younger generations, a generational analysis was conducted to explore the differences between Gen Z (born after 1996), Millennials (born between 1981 and 1996), Gen X (born between 1965 and 1981), and Baby Boomers (born before 1965). Descriptive statistics were computed for each mental health outcome for the overall sample and by Multiracial and multiethnic classification, generation, gender identity, sexual orientation, educational attainment, language spoken at home, and income.

Following overall descriptive statistics, simple logistic regression models were tested to explore the association between mental health outcomes and Multiracial and multiethnic group status, demographic variables, potentially traumatic events, experiences of unfair treatment, strength in ethnic identity, and multiethnic identity integration. Following an exploratory bivariate analysis of each sample, multivariable analyses were developed for Sample B, which had a sufficient sample size (*N* = 1,012). Significant variables from the bivariate analysis for each individual mental health outcome were added into a multivariable regression model one at a time; model fit was assessed using both AIC and BIC. The model with the best fit was retained.

Interview transcripts were analyzed using staged hybrid inductive-deductive thematic analysis using Dedoose (68). The research team created an initial codebook based on interview questions, which were based on the study questions of interest, *a priori* knowledge on the subject, and notes from the interviews. After three members of the study team piloted the codebook by coding two random transcripts from the first five interviews, the team met to discuss interpretation and adjust codes and definitions. Two members of the study team then double coded two transcripts and met to discuss any adjustments to the codebook. The final codebook was used by a primary coder who identified as Multiracial and multiethnic to code the remaining transcripts, which were reviewed by a secondary Multiracial/multiethnic coder. After coding was completed, the study team analyzed narratives using thematic analysis; common sentiments, domains, and themes were summarized and accompanied by illustrative quotes (Supplementary material S1). Subgroup analyses were conducted among subsets by racial and ethnic composition of the participants, gender identity, sexual orientation, and age. As part of the participatory approach, preliminary results were shared back with the interview participants; feedback was incorporated.

## 3 Results

### 3.1 Quantitative findings

Sample A (*n* = 347) was comprised of majority female-identifying (75.5%, *n* = 259), straight (63.5%, *n* = 214), and college graduates (71.7%, *n* = 246), with the majority reporting a household income of at least $60,000 (58.5%, *n* = 203). The majority of participants (71.1%, *n* = 244) were classified as having White & Non-White heritage, 28.9% (*n* = 99) were classified as having Non-White heritage. Almost over a quarter (28.0%, *n* = 97) of the sample reported having any Black or African American heritage, 37.2% (*n* = 129) any Hispanic or Latino heritage, 35.2% (*n* = 122) any Asian heritage, 13.0% (*n* = 45) any American Indian or Alaska Native heritage, 4.6% (*n* = 16) any Middle Eastern or North African heritage, 2.9% (*n* = 10) any Native Hawaiian or Pacific Islander heritage, and 21.3% (*n* = 74) heritage from another racial or ethnic group. About half (50.9%, *n* = 173) of participants were Millennials, 21.5% (*n* = 73) Gen-X, 16.2% (*n* = 55) Gen-Z, 11.2% (*n* = 39) Baby Boomers or prior generations (Table 1).

**Table 1:** Survey population demographics and unadjusted odds of at least one mental health condition.

	Sample A	Any mental health condition			Sample B	Any mental health condition		
		57.1% (198)				58.0% (587)		
	*N* = 347	OR	95% CI	*p*-value	*N* = 1,012	OR	95% CI	*p*-value
Multiracial category								
White/Non-White	71.1% (244)	1.00			55.0% (557)	1.00		
Non-White	28.9% (99)	1.07	[0.67, 1.72]	0.784	45.0% (455)	1.37	[1.06, 1.76]	0.015
Racial and ethnic identity								
White	71.5% (248)	0.92	[0.57, 1.47]	0.717	55.0% (557)	0.73	[0.57, 0.94]	0.015
Black or African American	28.0% (97)	1.10	[0.68, 1.77]	0.690	48.2% (488)	1.36	[1.06, 1.75]	0.016
Hispanic or Latino	37.2% (129)	0.92	[0.59, 1.43]	0.718	48.1% (487)	1.27	[0.99, 1.63]	0.064
American Indian or Alaska Native	13.0% (45)	2.60	[1.27, 5.33]	0.009	29.4% (298)	0.93	[0.71, 1.22]	0.590
Asian	35.2% (122)	0.83	[0.53, 1.29]	0.412	16.3% (165)	0.82	[0.59, 1.15]	0.248
Native Hawaiian or Pacific Islander	2.9% (10)	1.78	[0.45, 7.02]	0.408	8.5% (86)	1.18	[0.75, 1.86]	0.477
Middle Eastern or North African	4.6% (16)	2.34	[0.74, 7.40]	0.148	8.1% (82)	1.02	[0.65, 1.62]	0.919
Other	21.3% (74)	0.92	[0.55, 1.54]	0.746	8.9% (90)	1.15	[0.74, 1.79]	0.532
Educational attainment								
Less than college graduate	28.3% (97)	1.00			62.3% (627)	1.00		
College graduate +	71.7% (246)	0.41	[0.24, 0.67]	0.001	37.7% (379)	0.68	[0.53, 0.88]	0.003
Household income								
Less than $60 K	36.2% (115)	1.00			57.4% (552)	1.00		
$60 K +	63.8% (203)	0.67	[0.42, 1.06]	0.088	42.6% (409)	0.73	[0.57, 0.95]	0.019
Place of birth, respondent								
In the United States	84.1% (290)	1.00			88.1% (892)	1.00		
Puerto Rico or Other U.S. Territory	1.7% (6)	3.79	[0.44,32.83]	0.227	1.9% (19)	0.95	[0.38, 2.38]	0.909
Outside of the United States	13.6% (47)	0.94	[0.50, 1.74]	0.840	9.3% (94)	0.61	[0.40, 0.93]	0.022
Sexual orientation								
Straight	63.5% (214)	1.00			80.1% (798)	1.00		
Gay or Lesbian	5.0% (17)	2.23	[0.76, 6.54]	0.145	6.2% (62)	2.61	[1.46, 4.69]	0.001
Bisexual or something else	31.5% (106)	1.73	[1.07, 2.80]	0.025	13.7% (136)	4.71	[2.92, 7.59]	<0.001
Gender identity								
Male	19.0% (65)	1.00			30.1% (303)	1.00		
Female	75.5% (259)	1.94	[1.12, 3.36]	0.018	67.9% (683)	1.25	[0.95, 1.63]	0.115
Transgender or gender expansive	5.5% (19)	4.96	[1.48,16.57]	0.009	2.0% (20)	16.54	[2.19,125.10]	0.007
Generation								
Gen Z—1997+	16.2% (55)	2.74	[1.14, 6.57]	0.024	15.0% (152)	13.26	[7.56,23.27]	<0.001
Millennials—1981–1996	50.9% (173)	1.83	[0.88, 3.81]	0.108	43.0% (435)	4.57	[3.00, 6.95]	<0.001
Gen X—1965–1980	21.5% (73)	1.71	[0.76, 3.85]	0.197	27.4% (277)	3.19	[2.05, 4.95]	<0.001
Baby Boomers—1946–1964	10.3% (35)	1.00			14.0% (142)	1.00		
Perceived stress								
Low or no stress	37.8% (131)	1.00			26.1% (264)	1.00		
High levels of stress	62.2% (216)	7.92	[4.84,12.95]	<0.001	73.9% (748)	14.70	[10.15,21.29]	<0.001

Sample B (*n* = 1,012) was comprised of majority female-identifying (67.5%, *n* = 683), straight (80.1%, *n* = 798), attained less than a college degree (62.3%, *n* = 627), and a household income less than $60,000 (57.4%, *n* = 552). The majority of participants (55%, *n* = 557) were classified as having White and Non-White heritage, 45.0% (*n* = 455) as having Non-White heritage. Almost half of respondents (48.2%, *n* = 488) reported any Black or African American heritage, 48.1% (*n* = 487) any Hispanic or Latino heritage, 16.3% (*n* = 165) any Asian heritage, 29.4% (*n* = 298) any American Indian or Alaska Native heritage, 8.5% (*n* = 86) any Native Hawaiian or Pacific Islander heritage, 8.1% (*n* = 82) any Middle Eastern or North African Heritage, 8.9% (*n* = 90) heritage from a racial or ethnic group not listed. Almost half (43%, *n* = 435) of respondents were born between the years 1981–1996 and classified as Millennials; 27.4% (*n* = 277) between 1965 and 1980, classified as Gen-X; 15% (*n* = 152) after 1997, classified as Gen-Z; and 14% between 1946 and 1964, classified as Baby Boomers. Less than 1% of the sample were born before 1946 (Table 1).

#### 3.1.1 Exploratory analysis: mental health and social factors

**Figure 1:**
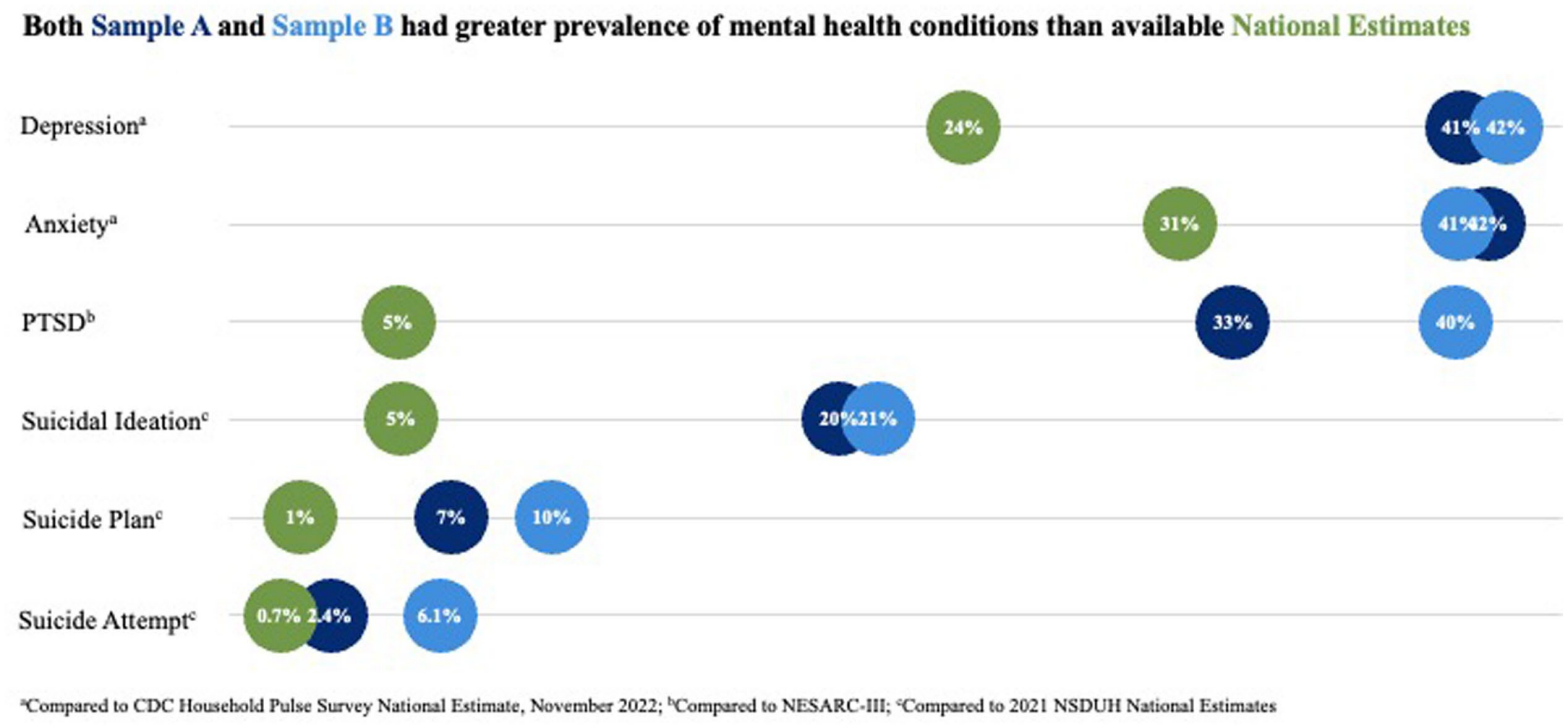
Both sample A and sample B had greater prevalence of mental health conditions than available national estimates. ^a^Compared to CDC Household Pulse Survey National Estimates, November 2022. ^b^Compared to NESARC-III. ^c^Compared to 2021 NSDUH National Estimates.

More than half of each Sample A (57.1%, *n* = 198) and Sample B (58.0%, *n* = 587) endorsed one or more clinically significant mental health concerns. Respondents endorsed clinically significant symptoms of depression (A: 40.6%, B: 42.1%), anxiety (A: 41.5%, B: 40.5%), PTSD (A: 32.9%, B: 40.4%), and STB (A: 21.1%, B: 25.4%) including suicide attempt (A: 2.4%, B: 6.1%) (Figure 1).

Differences in the unadjusted odds of endorsing symptoms for one or more mental health condition between White/Non-White and Non-White Multiracial and multiethnic groups were found in Sample B, with Non-White having greater odds as compared to White/Non-White (OR = 1.37, *p* = 0.015). The difference between these broad groups was not significant in Sample A. Bivariate analyses for Sample A suggest that participants with American Indian or Alaska Native heritage had significantly greater odds of having any mental health condition as compared to those with no heritage from that group (OR = 2.60, *p* = 0.009). This was not found in Sample B; however, unadjusted analyses for Sample B suggest increased odds of endorsing one or more mental health concern for respondents with any Black or African American heritage as compared to those without any, and decreased odds for those with any White heritage (OR = 0.73, *p* = 0.015) (Table 1).

Both samples found significantly reduced odds of endorsing one or more mental health condition for college graduates (A: OR = 0.41, *p* = 0.001; B: OR = 0.68, *p* = 0.003) as compared to those with less than a 4-year degree. Sample B had significantly reduced odds for people with a household income $60,000 or greater (OR = 0.73, *p* = 0.019); although the difference was not significant for Sample A, the data suggested a similar trend. People born outside of the U.S. in Sample B had significantly reduced odds of endorsing one or more mental health condition (OR = 0.61, *p* = 0.022); this difference was not significant in Sample A (Table 1).

As compared to straight respondents, Sample B found significantly increased odds of endorsing one or more mental health condition for people who identify as lesbian or gay (OR = 2.61, *p* = 0.001) and both samples found significantly increased odds of endorsing one or more mental health condition for people who identify as bisexual or something else (A: OR = 1.73, *p* = 0.025; B: OR = 4.71, *p* < 0.001). As compared to people who identify as male, Sample A found significantly increased odds for people who identify as female (OR = 1.94, *p* = 0.018) and both samples found significantly increased odds for people who identify as transgender or gender expansive (A: OR = 4.96, p = 0.009; B: OR = 16.54, *p* = 0.007) (Table 1).

As compared to Baby Boomers, both samples found significantly increased odds for Gen-Z respondents (A: OR = 2.74, *p* = 0.024; B: OR = 13.26, *p* < 0.001); Sample B also found significantly increased odds for Gen X (OR = 3.19, *p* < 0.001) and Millennials (OR = 4.57, *p* < 0.001). Both samples found significantly increased odds for respondents endorsing greater levels of stress (A: OR = 7.92, *p* < 0.001; B: OR = 14.70, *p* < 0.001) as compared to those with lower levels of stress (Table 1).

Both survey populations were exposed to a high volume of potentially traumatic experiences in their lifetime (A:0.8.3, SD = 4.3; B: 8.2, SD = 4.9), lifetime discrimination (A: 3.1, SD = 2.9; B: 3.6, SD = 3.3), everyday discrimination (A: 12.8, SD = 9.6, B: 15.4, SD = 11.9), and microaggressions (A: 20.1, SD = 11.4, B: 25.4, SD = 13.9). The majority of respondents in both samples reported high levels of perceived social support (A: 71.0%, B: 53.7%). Both samples reported similar levels of ethnic identity exploration (A: 20.2, SD = 5.4; B: 20.9, SD = 5.3), affirmation (A: 22.0, SD = 3.0; B: 21.8, SD = 3.8), and resolution (A: 11.3, SD = 3.4; B: 13.2, SD = 2.9), as well as multicultural identity categorization (A: 16.1, SD = 8.2; B: 16.1, SD = 8.7), compartmentalization (A: 26.3, SD = 12.9; B: 26.2, SD = 13.3), and integration (A: 30.6, SD = 11.0; B: 36.5, SD = 11.1) (Table 2).

**Table 2:** Survey population factors: potentially traumatic experiences, discrimination, social support, strength in ethnic identity and unadjusted odds of at least one mental health condition.

	Sample A	Any Mental health Condition			Sample B	Any Mental health Condition		
		57.1% (198)				58.0% (587)		
	*N* = 347	OR	95% CI	*p*-value	*N* = 1,012	OR	95% CI	*p*-value
PTEs, exposed	8.3 (4.3)	1.06	[1.01, 1.12]	0.016	8.2 (4.9)	1.18	[1.14, 1.21]	<0.001
PTEs, witnessed	2.7 (2.9)	1.00	[0.93, 1.08]	0.980	2.9 (3.3)	1.20	[1.14, 1.25]	<0.001
PTEs, experienced	3.9 (2.8)	1.18	[1.09, 1.29]	<0.001	4.0 (3.1)	1.24	[1.18, 1.30]	<0.001
Lifetime discrimination	3.1 (2.9)	1.19	[1.10, 1.30]	<0.001	3.6 (3.3)	1.30	[1.24, 1.37]	<0.001
Everyday discrimination	12.8 (9.6)	1.09	[1.06, 1.12]	<0.001	15.4 (11.9)	1.08	[1.06, 1.09]	<0.001
Microaggressions	20.1 (11.4)	1.04	[1.02, 1.07]	<0.001	25.4 (13.9)	1.06	[1.05, 1.07]	<0.001
Assumptions of inferiority	3.0 (3.1)	1.14	[1.06, 1.22]	0.001	4.5 (3.2)	1.22	[1.17, 1.27]	<0.001
Second-class citizen and assumption of criminality	1.8 (2.3)	1.16	[1.05, 1.28]	0.003	3.2 (2.8)	1.28	[1.22, 1.34]	<0.001
Microinvalidations	4.8 (3.3)	1.15	[1.07, 1.23]	<0.001	5.3 (3.1)	1.23	[1.17, 1.28]	<0.001
Exoticization and assumptions of similarity	4.2 (2.9)	1.17	[1.08, 1.27]	<0.001	5.1 (3.1)	1.26	[1.21, 1.32]	<0.001
Environmental microaggressions	4.9 (2.2)	0.94	[0.85, 1.04]	0.200	5.0 (2.3)	1.15	[1.09, 1.21]	<0.001
Workplace and school microaggressions	1.8 (1.9)	1.22	[1.08, 1.38]	0.001	2.4 (2.0)	1.41	[1.32, 1.51]	<0.001
Perceived social support								
Low	3.6% (12)	1.00			10.6% (107)	1.00		
Moderate	25.4% (86)	0.66	[0.13, 3.26]	0.610	35.8% (362)	0.38	[0.21, 0.69]	0.002
High	71.0% (240)	0.19	[0.04, 0.90]	0.036	53.7% (543)	0.11	[0.06, 0.21]	<0.001
Family								
Low	13.6% (46)	1.00			17.6% (178)	1.00		
Moderate	26.0% (88)	0.22	[0.08, 0.63]	0.004	32.1% (325)	0.54	[0.34, 0.85]	0.007
High	60.4% (204)	0.11	[0.04, 0.29]	<0.001	50.3% (509)	0.15	[0.10, 0.23]	<0.001
Friends								
Low	7.4% (25)	1.00			14.8% (150)	1.00		
Moderate	23.1% (78)	0.40	[0.13, 1.30]	0.129	36.7% (371)	0.37	[0.23, 0.59]	<0.001
High	69.5% (235)	0.20	[0.07, 0.60]	0.004	48.5% (491)	0.18	[0.11, 0.29]	<0.001
Significant other								
Low	3.8% (13)	1.00			9.0% (91)	1.00		
Moderate	13.6% (46)	0.65	[0.12, 3.45]	0.617	29.1% (294)	0.40	[0.21, 0.75]	0.005
High	82.5% (279)	0.20	[0.04, 0.93]	0.040	62.0% (627)	0.15	[0.08, 0.28]	<0.001
Ethnic identity scale								
Exploration	20.2 (5.4)	0.99	[0.95, 1.03]	0.647	20.9 (5.3)	1.00	[0.98, 1.02]	0.971
Affirmation	22.0 (3.0)	0.86	[0.79, 0.94]	0.001	21.8 (3.8)	0.85	[0.81, 0.89]	<0.001
Resolution	11.3 (3.4)	0.95	[0.89, 1.01]	0.097	13.2 (2.9)	0.92	[0.87, 0.96]	<0.001
Multiethnic identity integration scale								
Categorization	16.1 (8.2)	1.03	[1.00, 1.06]	0.025	16.1 (8.7)	1.03	[1.01, 1.04]	<0.001
Compartmentalization	26.3 (12.9)	1.04	[1.02, 1.06]	<0.001	26.2 (13.3)	1.03	[1.02, 1.04]	<0.001
Integration	30.6 (11.0)	0.99	[0.97, 1.01]	0.395	36.5 (11.1)	0.99	[0.98, 1.00]	0.089

Both samples found that increased exposure to potentially traumatic experiences (A: OR = 1.06, *p* = 0.016; B: OR = 1.18, *p* < 0.001), lifetime discrimination (A: OR = 1.19, *p* < 0.001; B: OR = 1.30, *p* < 0.001), everyday discrimination (A: OR = 1.10, *p* < 0.001; B: OR = 1.08, *p* < 0.001), and microaggressions (A: OR = 1.04, *p* < 0.001; B: OR = 1.06, *p* < 0.001) increased the odds of endorsing symptoms of one or more mental health condition, while high levels of perceived social support reduced the odds (A: OR = 0.19, *p* = 0.036, B: OR = 0.11, *p* < 0.001) when compared to low levels of perceived social support. Both samples also found that greater affirmation of ethnic identity (A: OR = 0.86, *p* = 0.001; B: 0.85, *p* < 0.001) reduced the odds, while greater categorization (A: OR = 1.03, *p* = 0.025; B: OR = 1.03, *p* < 0.001) and compartmentalization (A: OR = 1.04, *p* < 0.001; B: OR = 1.03, *p* < 0.001) of multicultural identities increased the odds (Table 2).

#### 3.1.2 Multivariable analysis: depression, anxiety, PTSD, and STB

Adjusting for age, educational attainment, sexual orientation, gender identity, potentially traumatic experiences, lifetime discrimination, microaggressions, perceived social support, and affirmation of ethnic identity,^1^ analyses of Sample B suggest an increase in the odds of depression, anxiety, PTSD, and STB for each generation as compared to Baby Boomers. Gen-Z had the greatest odds of depression (OR = 9.00, *p* < 0.001), anxiety (OR = 12.12, *p* < 001), PTSD (OR = 5.53, *p* < 0.001), and STB (OR = 8.63, *p* < 0.001). The odds of having depression (OR = 2.12, *p* = 0.001), PTSD (OR = 2.13, *p* = 0.002) or STB (OR = 1.83, *p* = 0.019) were significantly greater for those with high school graduation or less compared to those with a college degree or higher. The odds of having depression (OR = 1.87, *p* = 0.009), anxiety (OR = 1.76, *p* = 0.015), or STB (OR = 1.88, *p* = 0.009) were significantly greater for respondents who identify as bisexual or something else as compared to straight. The odds of having STB were also elevated for respondents who identify as lesbian or gay (OR = 2.59, *p* = 0.006) as compared to straight (Table 3).

**Table 3:** Adjusted odds of depression, anxiety, PTSD, or STB within a sample of multiracial/ethnic adults in the U.S.

	Depression, past 2 weeks			Anxiety, past 2 weeks			PTSD, past month			Suicidal thoughts and behaviors, past year			One or more mental health concern		
	OR	95% CI	*p*-value	OR	95% CI	*p*-value	OR	95% CI	*p*-value	OR	95% CI	*p*-value	OR	95% CI	*p*-value
Generation															
Gen Z—1997+	9.00	[4.62, 17.53]	<0.001	12.12	[5.97, 24.61]	<0.001	5.53	[2.83, 10.80]	<0.001	8.63	[3.58, 20.82]	<0.001	12.41	[6.18, 24.91]	<0.001
Millennials—1981–1996	3.56	[2.06, 6.16]	<0.001	5.53	[3.03, 10.07]	<0.001	2.67	[1.56, 4.58]	<0.001	4.15	[1.87, 9.24]	<0.001	4.04	[2.45, 6.66]	<0.001
Gen X—1965–1980	3.00	[1.70, 5.30]	<0.001	4.41	[2.37, 8.20]	<0.001	1.74	[0.99, 3.07]	0.056	2.78	[1.21, 6.37]	0.016	3.10	[1.85, 5.21]	<0.001
Baby Boomers-1946–1964	1.00			1.00			1.00			1.00			1.00		
Educational attainment															
High school graduate or less	2.12	[1.37, 3.29]	0.001	1.23	[0.80, 1.89]	0.356	2.13	[1.33, 3.39]	0.002	1.83	[1.11, 3.04]	0.019	1.83	[1.13, 2.95]	0.013
Some college	1.40	[0.99, 1.99]	0.058	1.06	[0.75, 1.51]	0.730	1.51	[1.04, 2.19]	0.031	1.40	[0.91, 2.15]	0.125	1.31	[0.92, 1.89]	0.138
College degree or higher	1.00			1.00			1.00			1.00			1.00		
Sexual orientation															
Straight	1.00			1.00			1.00			1.00			1.00		
Gay or Lesbian	1.05	[0.56, 1.99]	0.871	1.37	[0.73, 2.55]	0.328	0.84	[0.42, 1.68]	0.618	2.59	[1.32, 5.11]	0.006	1.29	[0.60, 2.75]	0.516
Bisexual or something else	1.87	[1.17, 2.99]	0.009	1.76	[1.12, 2.78]	0.015	1.55	[0.95, 2.50]	0.076	1.88	[1.17, 3.03]	0.009	1.91	[1.07, 3.40]	0.028
Gender identity															
Male	1.00			1.00			1.00			1.00			1.00		
Female	2.01	[1.41, 2.88]	<0.001	2.14	[1.50, 3.04]	<0.001	1.58	[1.09, 2.30]	0.016	1.26	[0.84, 1.89]	0.273	1.97	[1.36, 2.87]	<0.001
Transgender or gender expansive	4.95	[1.33, 18.43]	0.017	11.95	[2.33, 61.32]	0.003	46.08	[5.00,424.89]	0.001	2.88	[0.81, 10.24]	0.101	1.00		
Potentially traumatic experiences	1.08	[1.05, 1.12]	<0.001	1.09	[1.05, 1.12]	<0.001	1.16	[1.12, 1.21]	<0.001	1.07	[1.03, 1.12]	0.001	1.13	[1.09, 1.17]	<0.001
Lifetime discrimination	1.11	[1.04, 1.18]	0.002	1.14	[1.07, 1.21]	<0.001	1.13	[1.06, 1.21]	<0.001	1.08	[1.00, 1.16]	0.047	1.12	[1.04, 1.20]	0.003
Microaggressions	1.01	[1.00, 1.03]	0.047	1.01	[1.00, 1.03]	0.133	1.02	[1.00, 1.04]	0.014	1.03	[1.01, 1.04]	0.005	1.02	[1.01, 1.04]	0.002
Perceived social support															
Low social support	1.00			1.00			1.00			1.00			1.00		
Moderate social support	0.41	[0.24, 0.71]	0.001	0.57	[0.33, 0.96]	0.033	0.51	[0.29, 0.89]	0.018	0.67	[0.38, 1.19]	0.171	0.31	[0.15, 0.64]	0.002
High social support	0.23	[0.13, 0.39]	<0.001	0.31	[0.18, 0.52]	<0.001	0.25	[0.15, 0.43]	<0.001	0.47	[0.27, 0.83]	0.009	0.14	[0.07, 0.28]	<0.001
Affirmation of ethnic identity	0.95	[0.91, 1.00]	0.032	*			0.95	[0.91, 0.99]	0.025	0.92	[0.88, 0.96]	<0.001	0.91	[0.86, 0.97]	0.001
Intercept	0.18	[0.05, 0.66]	0.010	0.04	[0.01, 0.09]	<0.001	0.12	[0.03, 0.46]	0.002	0.11	[0.02, 0.49]	0.004	0.98	[0.20, 4.87]	0.978

*Item excluded from model.

There was a dose–response relationship between the odds of having depression, anxiety, PTSD, or STB with each additional potentially traumatic experience and lifetime discriminating event. There was also a dose–response relationship between each additional microaggression experienced and the odds of depression, PTSD, and STB. Greater perceived social support was protective of mental health conditions, with significantly greater odds of having depression, anxiety, PTSD, or STB among those with low social support as compared to high social support. Greater affirmation of one’s ethnic identity was associated with lower odds of having depression, PTSD, or STB. Odds ratios, 95% CIs, and *p*-values are available in Table 3. Models, AIC, and BIC are available in Appendix.

### 3.2 Qualitative findings

A total of 17 interviews were conducted and a codebook was developed to elucidate domains, themes, and subthemes (Supplementary material S1). Twelve of the Multiracial/multiethnic interviewees selected the racial/ethnic option for White; eight, Asian; seven, Black or African American; four, American Indian or Alaska Native; two, Hispanic or Latino. Eleven participants identified as female; four, male; two, gender expansive. Fourteen of the participants identified as straight and three as bisexual or another sexuality. Nine participants were Millennials; six, Gen X; one, Gen Z; and one, Baby Boomer.

#### 3.2.1 Experiences of mental health during childhood

Participants recalled a general absence of support for mental health from their family and community while growing up. Common sentiments about mental health included denial, avoidance, criticism, stigma, and dismissal of mental health challenges. Many participants reflected on their childhood and recognized experiences as being related to mental health that were not previously acknowledged, even when the mental health issue experienced was quite severe. When experiencing a mental health challenge, some participants were told it was not real and to ignore it. Others were explicitly forbidden from seeking mental health care by their family. In other extreme circumstances, people recall family members being “thrown away” or isolated from the family until the mental health issue was able to be ignored. For people who grew up in more socioeconomically disadvantaged environments, there was a general understanding that there were more pressing issues, such as meeting basic needs.

“Ignore it, avoid it, push it to the side, it will work itself out.”

When discussing cultural perceptions of mental health, participants unveiled several culturally specific terminologies. Many of the terms had spiritual or possession connotations, such as “possessed by a demon,” “being haunted,” and “seeing or hearing spirit” while others denoted a sense of brokenness, like “broken head.” There was a shared understanding among participants that employing such terms in a clinical setting could potentially lead to misunderstandings or adverse repercussions. Participants also shed light on various complementary mental health practices rooted in their cultures that were more positively received. These included seeking guidance from a traditional healer, the therapeutic act of journaling, and forging a deeper connection with nature.

#### 3.2.2 Experiences of mental health in more recent years

As adults, participants acknowledged the chasm that still exists between identifying the need for care and seeking help. Some participants came to the realization that experiences growing up may have been atypical or even harmful and contributed to internal turmoil. As adults who sought out mental wellness, participants reported concealing their mental health challenge from their family. For some, concealment aimed to avoid dialogue with family who still held stigmatizing beliefs. For others, concealment aimed to protect loved ones with untreated mental health issues.

“There’s no longer … it’s not anything to be ashamed of, it’s just this season if you’re going through a mental health crisis, and there’s plenty of services available. It’s a really nice shift.”

Participants reflected on the social changes in acceptance and normalization of mental health. Participants felt that people could now seek care and go to therapy, and that there are more processes and systems in place to support people going through a crisis. Participants reported working to overcome stigmatizing thoughts and beliefs they were raised with, while also recognizing their continued impacts. Participants reported seeking out communities of people with similar backgrounds and/or experiences to share resources and navigate the process of seeking wellness. However, there was a fear that new technology that allows police to access a person’s medical history would result in increased police brutality, particularly for children of color.

#### 3.2.3 Factors that impact mental health

Participants noted that fatigue, overworking, burden of responsibility, lack of support from one’s family, criticism from loved ones, isolation, and dehumanizing experiences exacerbated mental health challenges. Participants reported experiences of rejection by their racial/ethnic groups, local community, and their own family resulting in a complicated, confusing, and often isolating experience. As participants reported difficulty finding a natural fit within any one group, they reported utilizing strategies such as masking and code-switching to fit in with other groups and cope with the absence of belonging. Participants identified these strategies as additional mental health stressors and, importantly, noted the resulting impact these strategies had on concealing symptoms of mental health concerns. For some, this resulted in missed and/or late-in-life diagnoses of mental health and developmental conditions. Participants with additional marginalized identities, including sexual minorities, gender expansive people, and women, reported additional layers of isolation, experiences of violence, and challenges seeking and receiving safe care. These aspects of their intersectional identities were elevated as a higher priority when seeking out care, with safety being a main concern.

“An entire community can really be pushed to the brink by external stressors.”

#### 3.2.4 Experiences seeking out mental health support

Many participants experienced challenges trying to find support in recent years and reported having to go outside their local community to find care. Financial barriers were unanimously cited by individuals who sought and received care. This was the case for those with public, private, and insufficient insurance coverage. Provider availability was another large barrier. For those who were limited by their geographic area, there were few providers and the waitlists for those providers were several years. Increases in coverage for virtual care from providers outside of the geographic area reduced this barrier, but people continued to encounter long wait lists. Individuals also reported the challenges of lapses in care or coverage, particularly while experiencing a mental health crisis and attempting to navigate the various systems to get healthy.

“I am sometimes bound financially by the options afforded to me by my insurance provider.

And, a lot of times those people are overbooked”

The difficult and frustrating care seeking process had negative impacts on participants’ mental health and wellbeing. Participants also noted that finding a provider who could provide culturally appropriate care to be challenging, if not impossible, particularly for those living in less urban areas. Participants described a process that takes months or years to find an appropriate provider and having to begin the process again when they moved out of the city or state. Importantly, participants stressed the need to lower the barrier to entry for connecting to mental health care due to the absence of time, energy, or capacity to search for a provider that is available and affordable. Participants recommended that mental health providers provide additional information on their online profiles and sign up to appear on registries of providers serving diverse communities, and noted that they look specifically for providers whose profiles highlight the racial/ethnic background, gender identity, languages spoken, and sexuality of the provider; communities the provider has served and/or trained among; experiences with immigrants; explicit language highlighting that the provider is welcoming of LGBTQ+, neurodiverse, and disability community members.

“I’m tired and I don’t have the capacity all the time to just search and search forever.”

#### 3.2.5 Experiences with mental health providers

Many participants reported adverse experiences while receiving mental health care and identified the need to self-advocate. These adverse experiences included the application of inappropriate norms and standards, not feeling comfortable being open about harmful experiences perpetrated by the racial/ethnic group the provider is part of, being mishandled while experiencing a crisis, being completely excluded from the intake process, or being treated as “fascinating” by the provider. Participants also described experiences of the provider’s own microaggressions and bias, describing experiences of the provider openly attempting to classify them, expressing anti-miscegenation, actively misinterpreting the person’s words, and treating the person as less than human. For people with past traumatic experiences, experiences of being paired with a provider from a group that perpetrated the violence, or being put in a mixed group, was harmful. Participants with intersectional identities reported harmful experiences, with some comparing their past experiences to abusive relationships, experiencing fear of what the provider was writing down about them, having their experiences and realities dismissed, being given harmful advice, and even experiencing gaslighting by providers. As the social justice movement came back to the forefront over the past few years, participants noted experiences of providers performing social justice rather than providing culturally responsive care. For example, participants reported experiences where providers talked more about the J-DEI books they had read than apply knowledge from those books.

“I’m thankful that I have a voice and I speak the things that are injustices.”

Many participants had positive and beneficial experiences with mental health providers, while also noting that the negative experiences they encountered while finding the right provider. Experiences where a person was provided a care team that coordinated their services were seen as very positive, although infrequently experienced. For people who had been previously misdiagnosed or badly treated by mental health providers, a provider who included them in their assessment and care planning, while also acknowledging harm done to them, helped them to move forward. While many participants had positive sentiments about receiving care from a provider from their racial and/or ethnic community, few saw this as a possibility and fewer had worked with a provider sharing their racial or ethnic identity given the demographics of the provider population. Participants reported investing substantial time and effort in educating a provider on their cultural background, particularly the cumulative traumas experienced by Multiracial and multiethnic people. Participants reported sentiments of exhaustion and fatigue from having to bring providers up to speed and explain their existence in this world.

“People who don’t get what it means to have these

accumulative traumas inflicted on us every day.”

Participants expressed hope that finding a provider from their cultural community, or from any non-dominant community, would result in a more positive experience and less emotional labor. For those who had received care from a provider from a non-dominant community, the experience was typically recalled as quite positive. Providers who were from a similar community allowed a person to feel more at ease, not have to defend themselves, and not feel like the provider would automatically try to apply inappropriate norms and standards. Those with intersectional identities, particularly for participants who identified as queer, reported actively avoiding providers from their cultural community if that community was traditionally less accepting of queer identities. Others worried that the provider could have the same stigmatizing perspective they were raised. However, there was an overall desire to see more diversity among the provider population.

“I feel a connection with this person, like, I feel like I don’t have to spend the next 20 minutes explaining and articulating something that they probably wouldn’t understand anyways.”

#### 3.2.6 Peer recommendations for multiracial and multiethnic people seeking wellness

Participants were asked to provide recommendations for members of the Multiracial/ethnic community who may be struggling with mental health. Participants’ recommendations fell into three key categories: support, secure identity formation, and seeking care.

1. *“Know your support systems:”* Almost every participant recommended developing and leveraging chosen community and interpersonal supports. Participants reflected on the value of community support networks to access essential information, such as provider recommendations, online search criteria for providers, and registries of diverse providers. Participants described examples of these groups providing support for people with everyday experiences, experiences with mental health providers, unlearning stigmatizing beliefs, and general support navigating the world as a Multiracial and multiethnic person. Resources for and by people of color were cited as helpful. Participants also noted that there needs to be a crisis line by and for people of color to ensure the person is speaking to someone who can understand their experience. Participants also emphasized the need to differentiate between social support and social control, and to consider the validity of what is shared in online environments.
2. *“Be unwavering in who you are because society and the world at large will try to define you:”* Participants recognized the “tug of war” between cultural practices and beliefs and one’s identity. Participants recommended a person work toward self-acceptance and secure identity formation. Participants also recommended identifying and retaining the helpful aspects of culture, such as forming community and being in nature, while working past the harmful aspects, such as stigma and criticism. Participants recommended exploring positive coping and emotional regulation practices, with examples including adequate rest, work-life balance, being in nature, finding a good hobby, breathing practices, physical activity, and finding online community.
3. *“Do not ignore it. Start working on it. Go to therapy. You’re going to have different cultures that have told you different things, but it is not something that that you can ignore:”* Participants highlighted the need to reduce stigma of and normalize mental health. Therapy and finding someone to talk to was seen as beneficial. Participants noted that tools and strategies provided by therapists remain helpful during times they could not afford to see the provider. Participants advocated for trusting one’s instincts on the fit of the provider, investing the time to find a provider that is a good fit, and increasing one’s own knowledge of mental health. Participants noted the positive impact of educating themselves on their symptoms, diagnosis, and treatment options as well as known bias in the diagnostic classification systems. Given the challenges participants reported with the care-seeking process, participants recommended going back to support networks help curate and navigate the mental health landscape quickly. Participants also suggested the public health community increase the pool of culturally responsive providers for communities that experience greater stressors, such as poverty and food insecurity, and complementing this with school-based mental health education.

## 4 Discussion

This study provides crucial insights into the mental health status and experiences of Multiracial and multiethnic adults in the U.S., contributing to addressing significant gaps in our understanding of a population currently excluded and underrepresented in existing public health data infrastructure. The results highlight an urgent need to re-consider the approach of the public health community to prioritize J-DEI, particularly concerning mental health and wellbeing of populations excluded and/or underrepresented in routine public health infrastructure and practice, by (1) acknowledging and addressing systemic exclusion/erasure/underrepresentation of populations in existing public health data and practice, (2) identifying and addressing community-specific barriers to accessing high quality behavioral health care, (3) ensuring behavioral health providers are capable of providing culturally-responsive care to Multiracial and multiethnic populations while diversifying the pool of available providers, and (4) investing in community-developed and-based supports and approaches to care.

### 4.1 Health inequities and systemic racism

The findings suggest a high burden of mental illness among Multiracial and multiethnic adults, made evident by higher rates of depression, anxiety, PTSD, and STB when compared to other racial or ethnic groups and national estimates.

Compared to data for the Multiracial population in the CDC Household Pulse Survey conducted online around a similar time frame (November 2–14, 2022), both samples had a higher prevalence of depressive symptoms (CDC: 31.9%) and similar prevalence of symptoms of anxiety (CDC: 42.5%) (29). The CDC Household Pulse Survey is a representative sample, uses an Adapted PHQ-2 and Adapted GAD-2, does not include ethnicity when assessing for Multiracial status, and combines “other” with “multiple races,” which could explain some of these differences.

While PTSD is not a frequently collected population health metric in the U.S., the 2012–2013 National Epidemiologic Survey on Alcohol and Related Conditions III (NESARC-III) collected data for 12-month (4.7%) and lifetime (6.1%) PTSD (69). The proportion of Multiracial and multiethnic adults in our samples with clinically significant signs of PTSD in the prior 30-days is almost nine times greater and likely an underestimate of 12-month prevalence of PTSD in this sample. Studies assessing PTSD using NESARC-III data do not provide information for Multiracial or multiethnic adults; however, the highest prevalence found in the NESARC-III is among people identifying as American Indian or Alaska Native at 12.9%. The NESARC-III was conducted through face-to-face interviews more than 10 years ago, assessed PTSD using a narrow diagnostic definition tied to a specific potentially traumatic event, and assessed 12-month and lifetime PTSD; these methodological differences could explain some of the differences. Our larger study had a higher prevalence than the smaller study which could be partially explained by sampling processes, current events targeting Multiracial and multiethnic adults, multiple instances of racialized violence and violence against the LGBTQ+ community, and other potentially traumatic events happening in the U.S. during the study. As PTSD is not currently a routine health metric in the United States yet carries a high economic burden, we recommend public health practitioners consider including PTSD as a routine surveillance metric and that behavioral health clinicians incorporate trauma-responsive approaches, including PTSD screening tools, into their practice (70).

As with PTSD, the proportion of Multiracial and multiethnic adults from our two samples who had serious thoughts of suicide was more than four times greater and the proportion who had attempted suicide was almost nine times greater than national estimates from the 2021 NSDUH, which was predominantly Non-Hispanic White and found no significant racial or ethnic difference in STB (34). Our samples completed an anonymous online survey as compared to face-to-face household interviews which could partially explain the differences. As the U.S. is experiencing an increase in suicide rates among adolescents and some communities of color, these findings suggest behavioral health clinicians incorporate some element of screening for risk of suicide when working with Multiracial/ethnic clients (29).

The paucity of data on Multiracial and multiethnic populations within existing public health research, surveillance, and data infrastructure is indicative of systemic racism, perpetuating the exclusion, erasure, and marginalization of this growing population. The omission points to the dire need for comprehensive efforts to better understand and address the unique needs of Multiracial and multiethnic individuals, as well as other populations routinely excluded and/or underrepresented by existing structures, within the broader public health landscape. The increase in adjusted odds of depression, anxiety, PTSD, and STB with each subsequent generation following Baby Boomers is consistent with the existing literature describing worsening mental health among younger generations (71, 72). These findings, combined with the recent population estimates, highlight the urgency with which the public health community must adjust to include Multiracial and multiethnic populations.

### 4.2 Intersectionality and mental health

This study’s findings highlight the value of an intersectional lens in public health research and practice, recognizing the way gender identity, sexuality, race, and ethnicity can intersect to create unique vulnerabilities and/or strengths. Consistent with prior literature (73), this study found people who identify as bisexual or another expansive sexuality to have the greatest adjusted odds of depression, anxiety, and STB when compared to straight respondents. This study also found female and gender expansive respondents to have greater adjusted odds of depression, anxiety, and PTSD when compared to males (see Table 3 for detailed statistics).

Younger generations are more fluid in terms of gender and sexuality than prior generations and are becoming progressively more Multiracial and multiethnic. Scientific research must apply a multidimensional intersectional lens and updated analytic approaches to routine surveillance and in-depth investigations to adequately capture the health status of intersectional Multiracial and multiethnic adults and inform public health programming. Notably, as sexuality and gender are understood to be more of a spectrum than a categorical construct, there is a need to re-consider the current methodological approaches to ensure inclusion of expansive identities. In order to promote mental health equity, we must commit to understanding the unique needs of our diverse populations, reduce structural factors that exacerbate weathering, and provide a culturally responsive and safe healthcare delivery system (74). As social conditions continue to exacerbate stressors for people with diverse intersectional identities, there is a clear role for the public health community to strengthen protective factors and mitigate stressors. As our society becomes more diverse and strives to promote health equity, it is paramount that our research methodologies evolve to reflect this multifaceted reality. The results offer a call to action to not only recognize but also actively incorporate expansive identities in our research approaches.

Although not a focus of this study, we had a small number of transracial adoptees participate in this study, highlighting the complexity of identity for this population. We strongly recommend dedicated research into the health status of transracial adoptees given the increases in transracial adoption (75). We recommend that researchers be attentive racial disparities in the U.S.’ child welfare system and ongoing discussions on the realities and impacts of international adoption (76–78). Additionally, given advances in fertility options resulting in differences between biological and birth parenting status, and differences between the racial and ethnic identities of birth parents and children, we recommend researchers consider capturing these data in future research.

### 4.3 Protective factors, coping, and community support

Ongoing, routine, updated public health data on the mental health status of Multiracial and multiethnic people with samples robust enough to identify inequities across crucial social factors is a fundamental step; understanding factors that exacerbate and protect the wellness of people identifying as Multiracial and multiethnic must follow. Despite facing regular prejudice events, often from their own families or racial/ethnic communities, Multiracial and multiethnic adults demonstrate a strong sense of community. This study illuminates the complex environment Multiracial and multiethnic people navigate while seeking optimal wellness, similar to other communities of color (21, 39–41). This study provides evidence to suggest the Multiracial and multiethnic community experiences impacts from weathering: respondents with higher levels of stress had increased odds of each mental health outcome and increases in categorization or compartmentalization of multiethnic identity, potentially traumatic events, lifetime discrimination, everyday discrimination, and microaggressions had a dose–response relationship with increased odds of having a depression, anxiety, PTSD, or STB. Due to regular experiences of rejection from social groups, Multiracial and multiethnic adults developed strategies such as masking and code-switching as a means of coping which serves to conceal symptoms of mental illness, delaying diagnosis and treatment. This suggests a need to adjust clinical approaches for this population, which requires further investigation.

This study also identified indications of a protective effect from integration of multiethnic identities on depression and anxiety, and perceived social support on depression, anxiety, PTSD, and STB. These findings are consistent with research conducted among monoracial communities of color, yet provide unique information of the challenges and value of Multiracial/ethnic populations embracing their multiple racial and ethnic identities (14–16). The importance of family and community in understanding and addressing mental health may be important cultural values for certain groups (40, 79, 80). Despite reporting experiences of rejection from predominantly monoracial groups, participants reported re-engagement as these groups have become more accepting. This study finds Multiracial and multiethnic adults to be welcoming and supportive of community members, ready to support people who have struggled or are currently struggling through similar experiences. Multiracial and multiethnic people also emphasized the importance and value of secure identity formation, which created opportunity for them to embrace and accept their racial, ethnic, and cultural identities. The support structures described by study participants emphasize the importance of identity, community, and belonging. As literature suggests belonging to social groups, strength in ethnic identity, and multicultural ethnic identity integration can serve to protect against mental health issues, investment of resources to support Multiracial and multiethnic communities develop and maintain these assets is recommended (14–16).

### 4.4 Access to behavioral healthcare

The challenges faced by Multiracial and multiethnic individuals in accessing adequate behavioral health care accentuate the pressing need to bolster our behavioral health workforce. This involves not just increasing numbers but ensuring providers are trained in delivering culturally responsive care. This includes ensuring screening tools and diagnostic approaches account for masking and code-switching commonly utilized by Multiracial and multiethnic individuals. The challenges expressed by participants in receiving needed care highlight the impact of the behavioral health shortage, and support efforts to urgently build the behavioral health provider pool (81). There is a clear unmet need for available affordable mental health care, and an opportunity to recruit the growing population of Multiracial and multiethnic youth into the field. The potential of online community forums and virtual care models holds promise for reaching this diverse population, offering avenues for culturally sensitive, accessible care.

### 4.5 Opportunities to include underrepresented/excluded populations in public health research and practice

This study’s findings reveal the magnitude of the potential mental health burden and needs of Multiracial and multiethnic adults in the U.S. and demonstrate the importance of ensuring adequate representation of Multiracial and multiethnic populations in research and population surveys of mental health. As the U.S. reckons with the impact of racism as a public health crisis, the public health community must look inward at the systems and structures that serve to perpetuate inequities (82). As a complex arrangement or system of intentional and unintentional actions, structural racism is upheld by society through laws, social norms, and systems (83). This results in predictable patterns that allow public health workers to adjust for these patterns within surveillance, research, planning, and implementation.

#### 4.5.1 Critically appraising standard tools and instruments for population-fit

This study demonstrates the importance of ensuring tools and instruments are adequate for measuring health status and key risk and protective factors and social constructs within populations of Multiracial and multiethnic people. As the field of psychiatric epidemiology conducts rigorous psychometric testing on standard screening and diagnostic instruments for major racial and ethnic groups, this study highlights the importance of doing so for Multiracial and multiethnic populations. This must also be considered for tools designed to capture exposure to prejudice events and potentially traumatic experiences, as this study highlights the impact of exposure to these adverse experiences on the mental health of Multiracial and multiethnic adults. Furthermore, we strongly recommend the development of educational modules and clinical interventions specifically for Multiracial and multiethnic communities to both build the capacity of clinical, community and public health professionals to provide culturally responsive care for this growing population.

#### 4.5.2 A need to advance standards, routine practices, and social constructs

As national standards result in quite broad categories, there have been increasing calls to disaggregate these larger racial and ethnic groups as within-group differences are often masked, limiting the ability of the public health community to adequately identify and respond to health inequities. Localities with larger populations of diverse communities have recently demonstrated the need for this disaggregation: New York City, a city of 8.8 million, released a series of reports documenting the health differences within groups of Latino, Black, and Asian populations (84–86). As another example, there are numerous data issues when it comes to American Indian/Alaska Native communities including poor data quality due to not disaggregating data, several definitions of who is American Indian or Alaska Native, being excluded from data collection altogether (termed “data genocide” or “asterisk nation”), and lack of understanding of the political status held by tribal citizens of tribal nations that is separate from race or ethnicity (7, 87). These reports highlight clear differences in health outcomes experienced within each racial and ethnic group; however, the health of individuals who identify with multiple racial and/or ethnic groups is often unknown or presented with unreliable estimates, if at all.

Analyzing race and ethnicity data within Multiracial and multiethnic populations is complex as there are no established standards and Multiracial and multiethnic individuals have complex racial and ethnic backgrounds that make classification a multi-step process (4). As with other larger racial groups, within group disaggregation is essential to better understand the mental health needs of Multiracial and multiethnic populations. While the larger study did detect a difference between White/Non-White and Non-White Multiracial and multiethnic respondents, with Non-White Multiracial and multiethnic respondents having greater odds of endorsing one or more mental health concern, it did not find any significant difference when stratifying by each individual health outcome. These findings do not support those of Miller et al. (31) that found White/Non-White to have more depression symptomatology than aggregated Multiracial and monoracial groups. However, our study populations vary substantially given Miller et al. used the National Longitudinal Adolescent to Adult study (18–25) from 2001 to 2003, over two decades ago, and compared each Multiracial group to all Multiracial people. Additionally, our study included any person who identified as Multiracial and/or multiethnic and did not exclude Hispanic/Latino people from the analysis. Our study’s results by individual racial/ethnic group were inconclusive and warrant further investigation. For example, Sample A found significant differences for those identifying as American Indian or Alaska Native, which was not replicated in Sample B. While the two samples were collected in very different ways, and sample size for individual racial/ethnic groups limits our ability to adequately explore these findings, they do highlight the need for the field to go beyond exploratory research for this diverse and complex population and to ensure data can be disaggregated. Further research with current populations is needed to establish clinically and community meaningful classification procedures to standardize analyses, allow for comparability between studies, assess trends over time, and provide public health entities guidelines to implement routine public health surveillance within Multiracial and multiethnic populations.

#### 4.5.3 Acknowledging and accounting for exclusion in public health

Limited data on “smaller” demographic groups is a common challenge in public health. Current national standards for demographic data collection are a limiting factor. Established in 1997 and last updated in 2000, the standards include five categories for race (American Indian or Alaska Native, Asian, Black or African American, Native Hawaiian or Other Pacific Islander, White) and two categories for ethnicity (Hispanic or Latino, not Hispanic or Latino). Although current standards include the ability to select more than one race, actual practice does not ensure compliance with this standard and requires individuals and communities to self-classify themselves into existing groups that may be inadequate. These challenges have major implications, limiting the ability of the public health community to monitor and respond to unique health needs of Multiracial and multiethnic communities. Ensuring these standards evolve with changing populations to capture a sufficient spectrum of options can serve to affirm and respect the humanity and dignity of all people who have historically not been represented accurately and consistently in data.

As changing federal standards and public health data systems is a long process, there are ample opportunities for public health practitioners to assess, monitor, and address the health needs of their populations. This could include expanding the geographic reach of a study population, oversampling smaller populations, and working with organizations serving these excluded populations, to name a few. If population surveys are not able to oversample to have adequate coverage of Multiracial and multiethnic populations, there should be consideration given to the development of a representative survey on Multiracial and multiethnic mental health. As these recommendations require time and resources, this study leveraged PAR, convenience sampling, and mixed methods to explore the health status of a population in a way that could be easily and efficiently replicated by any public health team interested in better understanding the health status and needs of an underrepresented population to reduce delays and gaps in achieving health equity.

It is important to note that this study excluded people without high English reading levels or people unable to access an online survey. Language, literacy, and ability to access written text are critical structural barriers that limit inclusivity and have the potential to exacerbate health inequities. While this study relied on convenience samples and attempted to utilize a rapid PAR approach to highlight potential health inequities in an underrepresented/excluded population with minimal resources, it inherently excluded some of this population. As technology and virtual environments become more accessible and survey vendors adopt more inclusive recruitment strategies, there will be ample opportunities for researchers to conduct a similar study on a similar timeline with more inclusive approaches. It is critical that the field advocate for and leverage these advancements as we work to reimagine our systems, practices, and approaches (88).

## Data availability statement

The datasets presented in this article are not readily available because the participants of this study did not give written consent for their data to be shared publicly. With permission by the Johns Hopkins University IRB, an anonymized dataset from the quantitative study can be made available upon written request to the corresponding author. Due to the sensitive nature of the research, supporting data from the qualitative study are not available. Requests to access the datasets should be directed to JS, jshaff1@jhu.edu.

## Ethics statement

The studies involving humans were approved by the Johns Hopkins University Institutional Review Board. The studies were conducted in accordance with the local legislation and institutional requirements. Participants in the quantitative studies provided non-identifying written consent; participants in the qualitative study provided verbal consent.

## Author contributions

JS: Conceptualization, Data curation, Formal analysis, Funding acquisition, Investigation, Methodology, Project administration, Writing – original draft. XW: Data curation, Formal analysis, Writing – review & editing. JC: Formal analysis, Writing – review & editing. SB: Methodology, Writing – review & editing. HW: Conceptualization, Funding acquisition, Methodology, Project administration, Supervision, Writing – review & editing.

## Footnotes

^1^Affirmation of ethnic identity worsened model fit for anxiety and was dropped from the model.
