# Supplementary material for: Mental health and Multiracial/ethnic adults in the United States: a mixed methods participatory action investigation
Source: Front Public Health. 2024 Jan 11;11:1286137. doi: 10.3389/fpubh.2023.1286137 (PMC10808380; doi:10.3389/fpubh.2023.1286137)
Supplement: Supplementary file 1 [file Table_1.pdf]

Supplement A. Qualitative Codebook

Domain	Parent Code	Child Code	Description	Sample Quotation
**Growing up - experiences of mental health**			**Experiences of mental health and wellness for themselves and others as they were growing up.**	**" That's a hard one because I'm, looking back now I'm sure we experienced a lot of mental health issues – sadness, grief, depression. I don't think we handled it the best way possible or had the best tools available to us. "**
	Cultural elements of mental health		Words, descriptions, or phrases that were used to describe things related to mental health that are specific to the person's culture and/or family.	"iIt was like you need to pray to God about it, that was the only way to get past it or to overcome the mental illness not that it was a life challenge, or something that possibly is not going to get prayed away, you know. "
	Awareness of self or family experiencing mental health issue while growing up		Person affirmed that they or someone close to them experienced a mental health challenge while the person was growing up and describes their awareness and experience of it as a mental health issue at the time.	"I had a lot of mental health things when I was growing up, but I don't think any of my family recognized it."
	Sentiments about support		The person shares sentiments or memories about support available for mental health issues, while they were growing up. Support may be non-clinical in nature, and could include tools and resources	"There was not a lot of discussion. It's like people were, it's either the 95% of people who were normal, or there was, you know, people who are crazy. And there was nothing in between those. There was no, you know, no concepts of self-care or, you know, dealing with trauma and stuff."
	Stigmatizing experiences		The person describes stigmatizing experiences related to mental health and mental health support that they recall from when they were growing up.	"there was never, never any real discussion about mental health. Everybody was always capable of picking themselves up by their bootstraps and pushing through, regardless of the chaos."
**Recent years - experiences of mental health**			**Experiences of mental health and wellness for themselves and others in more recent years.**	**"Things have changed so much. Nowadays, with the campaign…the national, you know, global campaign for mental wellness, people can openly discuss the importance of self-care and getting help when it's needed and that it's OK to need help and that's part of life. There's been a huge shift. There's no longer…it's not anything to be ashamed of, it's just this season if you're going through a mental health crisis, and there's plenty of services available. It’s a really nice shift."**

Domain	Parent Code	Child Code	Description	Sample Quotation
	Approaches to mental health from childhood to adulthood		The person describes changes, differences, or similarities in approaches to mental health in adulthood as compared to childhood.	"growing up, if I had been going through like something similar, I don't think they would have necessarily, like, counselor or therapist would not have been their first thought."
	Experiences receiving mental health support		The person describes experiences with or feelings about support (availability, seeking, receiving, etc.) for mental health challenges, as an adult.	"Earlier this year I had I had a suicide attempt and it was very different from what I experienced or I saw, I guess, as a kid insofar as, like, I've really leaned into, like, western understanding of mental health and how to treat it "
	Barriers in accessing high quality care		The person describes barriers that impact access to high quality care.	"I am sometimes bound financially by the options afforded to me by my insurance provider. And, a lot of times those people are overbooked"
**Attributes of mental health care**			**Perspectives on experiences with mental health care in more recent years.**	**"I've definitely have had to fight doctors, like, to get the proper, like, just even to get them to answer my questions or to like feel like I'm being heard."**
	Attributes of culturally appropriate & responsive care		The person describes different parts and experiences of mental health care that are impacted by their racial, ethnic, and cultural background.	" that mental tug of war that I'm sure, you know, I mean like all of us who grew up in this country who have parents who are not from here, we all recognize that, we all can feel that and it definitely shaped our mental health, it shaped our world, it shaped who we are, and it's hard to talk to someone about yourself if they don't get that. "
	Opportunities for non'-'minority providers to improve care for minorities		The person describes strategies, approaches, tools, etc. for providers from the dominant group to improve care for people who are not from the dominant group	"a lot of it has to do with where the provider is providing those services. Like at Phoenix Indian Medical Center, you know, that's a group of special providers. They're very, very special providers because they're immersed in cultures, you know, that they travel across the state to come, you know, they're immersed in it. And it's a different level of patient care, and I noticed the difference."
	Sentiments about racial/ethnic concordance		The person shares thoughts of and/or experiences with receiving care from a provider whose racial and/or ethnic background is more similar to their own; this does not have to be perfect concordance, but could include care by a provider not from the dominant group. This can include seeking care.	"it would make sense for someone to assume that the quality of your experience may be better, right, talking to someone who has an idea of where you come from and what your values are and can relate to you culturally, and doesn't require a lot of explanation, who kind of just gets it - I think absolutely the quality of the experience could be could be better."

Domain	Parent Code	Child Code	Description	Sample Quotation
**Factors that impact mental health**			**Factors identified by participants as having an impact on mental health, mental health care, and wellness.**	**"Access to care, yes. And especially when people are in a poverty situation where they're going to have a lot of external stressors, that's exactly when people need services more available, not less."**
	Colorism		Describes how the shade, tone, or color of a person's skin may impact their mental health, care, etc.	"I just hate it. It has made my life double hard. It has made my attempt to be what I want to be double hard. Because I don't care how much education you get and much credentials you get behind your name, your color is going to always be there."
	Gender identity		Describes how a person's gender identity may impact their mental health, care, etc.	"I am probably nonbinary, probably agender, which is, of course, the most chill of all genders, except for being, you know, slightly unusual. So, while, this doesn't affect me a great deal, and this is something that, eh, I do prefer my - I guess, yeah, the LGBT friendly is probably a stronger thing for me than specific disabilities or specific ethnic things at all. "
	Sexual orientation		Describes how a person's sexuality or sexual orientation may impact their mental health, care, etc.	"bi folks, we have the worst mental health outcomes in terms of, like, measured by depression or anxiety outcomes than both gay and straight folks. And much of that is attributed to the fact that we are not fully in-group in either one of those. "
	Socioeconomic status, perceived or otherwise		Describes how a person's socioeconomic status (income, wealth, education, occupation, where they live) may impact their mental health, care, etc.	" I noticed, myself, that people's education levels, when it comes across verbally, how they speak seems to be what determines more…how much help they'll receive, based on articulation, honestly. "
	Neurodiversity and other disability status		Describes how a person's neurodiversity (ASD, ADHD, etc.) or other disability status may impact their mental health, care, etc.	"This has backfired on me a bit - there was one clinician who had some very strong expectations of what I was looking for as a male autistic person."
	Lack of acceptance by community		Describes the impact of lack of acceptance by a community or communities.	"a lot of people come from wherever, come to New York because they know they can find home here they can find community here and, you know, just talking with friends and colleagues like the source of grief and the source of turmoil and the source of sadness comes from their families from wherever they're from. "

Domain	Parent Code	Child Code	Description	Sample Quotation
	Third Culture Kid		The person grew up between cultures, oftentimes outside of the United States for some portion of childhood, and reflects on experiences related to navigating multiple different cultural dynamics.	"it was kind of a shock to come to North America and be, like, oh I'm, like, I'm just Black here, cool."
	Pressure of acculturation		The person describes the impact of the pressure to acculturate to the United States.	" there's social norms and there's even masks that people wear, a lot of times, to assimilate. "
	Exacerbators of mental health issues		Describes elements, factors, etc. that make mental health worse.	"Getting tired is the big one, but not just a little bit sleepy, but also worn out from stuff and just, as I've dealt with anxiety and depression, the big thing has been stuff that used to fill my tanks and leave me energized is just draining me. "
**Recommendations for people seeking wellness**			**Participants reflect on advice they would give to multiracial and multiethnic people struggling with their mental health.**	**" I think once you've normalized the being the feeling of not being OK, I think it opens up a lot of windows for you and a lot of opportunities that, like, seeking help is normal, seeking help is fine"**
	Access to culturally responsive providers with availability		Describes the benefits and challenges with finding culturally responsive mental health providers.	" I think especially if the mental health challenge is tied to questions about ethnicity and identity, like, feeling like I don't really have anyone to talk to about that who understands it. "
	Impact of social and interpersonal support		Describes recommendations to seek out and develop social and interpersonal support, online and in person.	"community is a big one because I feel like once that happens you can really branch out into like feeling more like confident or more supported"
	Benefits of mental health care		Describes recommendations related to receiving mental health care.	"Don't ignore it. Start working on it. Go to therapy. You're going to have different cultures that have told you different things, but it is not something that that you can ignore."
	Importance of secure identity formation and self'-'advocacy		Describes recommendations related to understanding one's own true identity and the importance of being able to self-advocate.	"#1, just be honest. Be honest and upfront with yourself and whoever you choose to talk to. I would say #2 trust your own instincts with choosing a professional, because whether or not you click with the person is really important in whether or not you can build trust. "

Domain	Parent Code	Child Code	Description	Sample Quotation
		Accepting all parts of oneself or accepting one's full self	Describes the ways a person accepts who they are and their full identity; includes unlearning stigmatizing beliefs or thoughts that they have about mental health, in order to improve their own mental health and be their full self.	"Be unwavering in who you are because society and the world at large will try to define you in 1001 ways"
		Benefits of fostering positive coping and emotional regulation practices	Describes the ways a person finds healthy ways to help their own mental health.	"Breathing practices that reduces stress. Breathing practices that brings you closer to a clarity - it slows down the body, slows down the mind, so maybe it'll give you some some breath to think. So that way your reactions aren't so rash, aren't so on the surface. "
