# Supplementary material for: Mental health and Multiracial/ethnic adults in the United States: a mixed methods participatory action investigation
Source: Front Public Health. 2024 Jan 11;11:1286137. doi: 10.3389/fpubh.2023.1286137 (PMC10808380; doi:10.3389/fpubh.2023.1286137)
Supplement: Supplementary file 2 [file Data_Sheet_1.pdf]

Glossary of Terms

- **Cisgender:** “an individual whose gender identity aligns with their sex assigned at birth.”[5]
- **Colorism:** “prejudice or discrimination especially within a racial or ethnic group favoring people with lighter skin over those with darker skin”[1]
- **Discrimination:** “the unjust and differential treatment of the members of different age, gender, racial, ethnic, religious, national, ability identity, sexual orientation, socioeconomic, and other groups at the individual level (e.g., behavioral manifestation of prejudice involving negative, hostile, and injurious treatment of the members of targeted groups) and the institutional/structural level (e.g., operating procedures, laws, and policies) that favor certain groups over others and has the effect of restricting opportunities for other groups.” [5]
- **Ethnicity:** “a characterization of people based on having a shared culture (e.g., language, food, music, dress, values, and beliefs) related to common ancestry and shared history.” [5]
- **Gender identity:** “How an individual identifies in terms of their gender. Gender identities may include, “male,” “female,” “androgynous,” “transgender,” “genderqueer” and many others, or a combination thereof.”[2]
- **Gender:** “the socially constructed roles, behaviors, activities, and attributes that a given society considers appropriate for different genders. In a human context, the distinction between gender and sex reflects the usage of these terms: Sex refers to the biological status of being male, female, or intersex, whereas gender implies the psychological, behavioral, social, and cultural aspects of gender (i.e., masculinity, femininity, nonbinary, nonconforming, or other gender).” [5]
- **Intersectionality:** “the complex, cumulative way in which the effects of multiple forms of discrimination (such as racism, sexism, and classism) combine, overlap, or intersect especially in the experiences of marginalized individuals or groups”[1]
- **LGBTQ+:** “acronym for lesbian, gay, bisexual, transgender, and questioning or queer: an inclusive term used to refer to the diverse forms of gender identity and sexual orientation, and to those whose gender identity differs from the culturally and socially determined gender roles for their assigned sex. More commonly seen as LGBTQ+, to recognize those not captured within or represented by the acronym LGBTQ.” [5]
- **Microaggressions:** “commonly occurring, brief, verbal or nonverbal, behavioral, and environmental indignities that communicate derogatory attitudes or notions toward a different “other.” Microaggressions may be intentional or unintentional, and the perpetrators may possibly be unaware of their behavior.” [5]
- **Multiracial/multiethnic:** National standards for racial and ethnic data collection set by the federal government include five categories for race (American Indian or Alaska Native (AI/AN), Asian, Black or African American, Native Hawaiian or Other Pacific Islander, White, Other) and two categories for ethnicity (Hispanic or Latino, not Hispanic or Latino).[3] To the authors’ knowledge, most public health entities and scientific research studies do not incorporate Hispanic and Latino populations within their definition of Multiracial. As this study includes Hispanic and Latino populations as well as populations identifying as “Middle Eastern or North African,” this study refers to the sample population broadly as Multiracial and/or multiethnic. Racial categories are socially constructed and have changed throughout history.[4,6] Please note that this approach to gathering racial/ethnic background does not fully or adequately capture the

rich diversity of the study population, nor the language used by each individual participant to describe their background.

- **Queer:** "An umbrella term used to describe a sexual orientation, gender identity or gender expression that does not conform to dominant societal norms. While it is used as a neutral, or even a positive term among many LGBT people today, historically "queer" was used as a derogatory slur."[2]
- **Race:** "the social construction and categorization of people based on perceived shared physical traits that result in the maintenance of a sociopolitical hierarchy."[5]
- **Sexual Orientation:** "a person's sexual and emotional attraction to another person and the behavior and/or social affiliation that may result from this attraction." [5]
- **Sexuality:** "the capacity to derive pleasure from various forms of sexual activity and behavior, particularly from sexual intercourse." [5]
- **Social support:** "the provision of assistance or comfort to others, typically to help them cope with biological, psychological, and social stressors. Support may arise from any interpersonal relationship in an individual's social network, involving family members, friends, neighbors, religious institutions, colleagues, caregivers, or support groups. It may take the form of practical help (e.g., doing chores, offering advice), tangible support that involves giving money or other direct material assistance, and emotional support that allows the individual to feel valued, accepted, and understood."[5]
- **Socioeconomic status:** "Encompasses not only income but also educational attainment, occupational prestige, and subjective perceptions of social status and social class. SES encompasses quality-of-life attributes and opportunities afforded to people within society and is a consistent predictor of a vast array of psychological outcomes." [5]
- **Stress:** "the physiological or psychological response to internal or external stressors. Stress involves changes affecting nearly every system of the body, influencing how people feel and behave." [5]
- **Systemic racism:** "forms of racism that are pervasively and deeply embedded in and throughout systems, laws, written or unwritten policies, entrenched practices, and established beliefs and attitudes that produce, condone, and perpetuate widespread unfair treatment of people of color."[6]
- **Transgender:** "an umbrella term used to describe the full range of people whose gender identity and/or gender role do not conform to what is typically associated with their sex assigned at birth." [5]

---

[1] Merriam-Webster Dictionary. https://www.merriam-webster.com/dictionary/
[2] GLSEN Key Concepts and Terms. https://www.glsen.org/sites/default/files/2020-04/GLSEN%20Terms%20and%20Concepts%20Thematic.pdf
[3] Office of Management and Budget. Revisions to the Standards for the Classification of Federal Data on Race and Ethnicity. (1997) https://www.govinfo.gov/content/pkg/FR-1997-10-30/pdf/97-28653.pdf
[4] Atkin AL, Christophe NK, Stein GL, Gabriel AK, Lee RM. Race Terminology in the Field of Psychology: Acknowledging the Growing Multiracial Population in the US. *Am Psychol* (2022) 77:381–393. doi: 10.1037/amp0000975
[5]American Psychological Association Dictionary of Psychology. https://dictionary.apa.org/
[6] Braveman, P. A., Arkin, E., Proctor, D., Kauh, T., & Holm, N. (2022). Systemic And Structural Racism: Definitions, Examples, Health Damages, And Approaches To Dismantling. *Health affairs (Project Hope)*, *41*(2), 171–178. https://doi.org/10.1377/hlthaff.2021.01394
